# Strain Modulation of Selectively and/or Globally Grown Ge Layers

**DOI:** 10.3390/nano11061421

**Published:** 2021-05-28

**Authors:** Yong Du, Guilei Wang, Yuanhao Miao, Buqing Xu, Ben Li, Zhenzhen Kong, Jiahan Yu, Xuewei Zhao, Hongxiao Lin, Jiale Su, Jianghao Han, Jinbiao Liu, Yan Dong, Wenwu Wang, Henry H. Radamson

**Affiliations:** 1 Key laboratory of Microelectronic Devices & Integrated Technology, Institute of Microelectronics, Chinese Academy of Sciences, Beijing 100029, China; duyong@ime.ac.cn (Y.D.); xubuqing@ime.ac.cn (B.X.); kongzhenzhen@ime.ac.cn (Z.K.); yujiahan@ime.ac.cn (J.Y.); zhaoxuewei@ime.ac.cn (X.Z.); linhongxiao@ime.ac.cn (H.L.); sujiale@ime.ac.cn (J.S.); hanjianghao@ime.ac.cn (J.H.); liujinbiao@ime.ac.cn (J.L.); dongyan2019@ime.ac.cn (Y.D.); wangwenwu@ime.ac.cn (W.W.); 2 Institute of Microelectronics, University of Chinese Academy of Sciences, Beijing 100049, China; 3 Research and Development Center of Optoelectronic Hybrid IC, Guangdong Greater Bay Area Institute of Integrated Circuit and System, Guangzhou 510535, China; liben@giics.com.cn; 4 CAS Key Laboratory of Quantum Information, University of Science and Technology of China, Hefei 230026, China; 5 Department of Electronics Design, Mid Sweden University, Holmgatan 10, 85170 Sundsvall, Sweden

**Keywords:** Ge, compressive, tensile, selective epitaxial growth (SEG), strain, RPCVD

## Abstract

This article presents a novel method to grow a high-quality compressive-strain Ge epilayer on Si using the selective epitaxial growth (SEG) applying the RPCVD technique. The procedures are composed of a global growth of Ge layer on Si followed by a planarization using CMP as initial process steps. The growth parameters of the Ge layer were carefully optimized and after cycle-annealing treatments, the threading dislocation density (TDD) was reduced to 3 × 10^7^ cm^−2^. As a result of this process, a tensile strain of 0.25% was induced, whereas the RMS value was as low as 0.81 nm. Later, these substrates were covered by an oxide layer and patterned to create trenches for selective epitaxy growth (SEG) of the Ge layer. In these structures, a type of compressive strain was formed in the SEG Ge top layer. The strain amount was −0.34%; meanwhile, the TDD and RMS surface roughness were 2 × 10^6^ cm^−2^ and 0.68 nm, respectively. HRXRD and TEM results also verified the existence of compressive strain in selectively grown Ge layer. In contrast to the tensile strained Ge layer (globally grown), enhanced PL intensity by a factor of more than 2 is partially due to the improved material quality. The significantly high PL intensity is attributed to the improved crystalline quality of the selectively grown Ge layer. The change in direct bandgap energy of PL was observed, owing to the compressive strain introduced. Hall measurement shows that a selectively grown Ge layer possesses room temperature hole mobility up to 375 cm^2^/Vs, which is approximately 3 times larger than that of the Ge (132 cm^2^/Vs). Our work offers fundamental guidance for the growth of high-quality and compressive strain Ge epilayer on Si for future Ge-based optoelectronics integration applications.

## 1. Introduction

Ge has long been desired as a suitable candidate to overcome the physical limits of conventional Si-based device structures [1,2,3]. To continue making full use of the traditional Si CMOS and reduce the costs of chips, epitaxial growth of high-quality Ge layer on Si has dramatically attracted attention. This approach is created due to the potential of Ge for optoelectronics applications, such as low-threshold Ge lasers [4,5], high-performance Ge photodetectors [6,7], high-performance Ge modulators [8,9], and high-mobility Ge electronic devices [10,11,12], etc. Furthermore, Ge buffer layers can also be regarded as a feasible platform for the growth of large lattice mismatch materials such as GaAs [13,14,15], InP [16,17], GeSn [18,19,20] on Si, which makes other novel optoelectronic devices on Si possible. 

However, the growth of high-quality Ge on Si faces the difficulty of large lattice mismatch and large thermal expansion coefficients between Ge and Si. To decrease the threading dislocation densities (TDDs) and surface roughness of the Ge layers, several methods have been proposed, such as introducing a Ge buffer of low-temperature (LT) and high-temperature (HT) growth [21,22,23,24], As-doped LT-Ge buffer [25], ultra-thin SiGe/Si superlattice buffer layer [26], reversed graded SiGe buffer [27], high-temperature H_2_ annealing [28,29], cyclic thermal annealing [30], and selective epitaxial growth (SEG) of Ge buffer [31]. By using the above-mentioned methods, material quality for Ge epilayers has significantly improved. However, the Ge buffer layers after post-annealing suffer from tensile strain, which emerges from the thermal expansion cooling process of Si and Ge. Therefore, it is very challenging to grow compressive Ge layers on Si for pMOS channel material. 

This work presents novel epitaxial methods to modulate the defect density and strain, and as a result, the PL property of Ge layers is improved. The growth morphology has been investigated in the following parts: In part 1, we study the growth mechanism of SEG Ge in the patterned Ge-on-Si substrate, and the quality of SEG Ge was verified by high-resolution transmission electron microscopy (HRTEM) analysis. In part 2, we evaluate the Ge strain at different positions in the grown Ge layers by HRXRD and HRTEM tools. In addition, the evolution of strain from tensile to compressive and its mechanism are systematically studied. The outcome of these novel processes provides an understanding for inducing strain and its mechanism for future Ge-based photoelectric devices.

## 2. Materials and Methods

**Figure 1:**
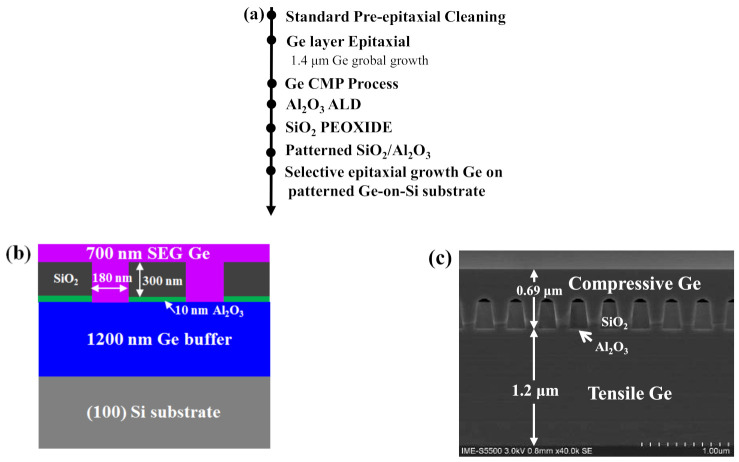
(**a**) Main process flow diagram; (**b**) material structure; (**c**) cross-sectional SEM of the SEG Ge on patterned Ge/Si structure.

In this study, all the Ge layers were grown on the 8-inch *p*-type Si (100) wafers with resistivity of 0.5–100 Ohm cm. The growth procedure is divided into several steps: (i) tensile-strained Ge growth using (a two-step method) LT-HT profile; (ii) CMP and patterned SiO_2_ fabrication; (iii) SEG of Ge top layer using HT-Ge growth condition (growth details are given in the experimental part). Firstly, a 1.4 μm Ge layer was deposited on the Si (100) wafer using a two-step growth in a reduced pressure chemical vapor deposition (RPCVD) reactor (ASM Epsilon 2000, Almere, The Netherlands). Germane (GeH_4_) diluted in H_2_ was used as a Ge precursor. Details of the growth chamber, including flux and substrate heater calibration, are described elsewhere [24]. After the Ge buffer growth, chemical mechanical polishing (CMP) was applied to ensure a smooth surface. Secondly, 10 nm Al_2_O_3_ layer was deposited by atomic layer deposition (ALD) reactor (TFS200, Beneq, The Netherlands) on the Ge layer and then 300 nm thick SiO_2_ was deposited on the Ge-on-Si substrate using plasma-enhanced CVD (PECVD) reactor (D250L, Corial, France) with SiH_4_ and N_2_O. In this stage, SiO_2_ and Al_2_O_3_ were then patterned into trenches along (110) with 180 nm and 220 nm width arrays. A 300 nm depth of vertical oxide sidewall profile was made using conventional DUV photolithography and reactive-ion etching (RIE). Thirdly, before the Ge selective epitaxial growth (SEG), the patterned Ge-on-Si substrates were immersed into BOE (49 wt% HF and 40 wt% NH_4_F with volume ratio of 1:7) for 2 min to remove the natural oxide. As high HF concentrations can damage the adhesion of Ge and SiO_2_, the BOE solution was mixed with deionized water at a ratio of 1:100, and the samples were rinsed in deionized water for 1 min. The SEG of Ge was performed at 650 °C in a partial pressure of 20 Torr providing a growth rate of 1.77 nm/s. Figure 1a–c displays the main process flow and the manufacturing steps of SiO_2_ channel and the selective growth (SG) Ge layer in this study.

Cross-section morphology was analyzed by scanning electron microscopy (SEM) HITACHI 5500 Japan. Atomic force microscopy (AFM) Bruker DIMENSION ICON was used to measure surface roughness. The samples were also characterized by high-resolution transmission electron microscopy (HRTEM) to determine the crystalline quality and the strain distribution. TEM specimens were picked up from target areas in the coalesced Ge layers by focused Ga ion beam (FIB microsampling method) and then polished in an ion milling system using Ar ion. Furthermore, energy-dispersive spectroscopy (EDS) was employed to determine the element materials of Ge layers. High-resolution X-ray diffraction (HRXRD) and high-resolution reciprocal lattice maps (HRRLMs) were used to measure the strain changes in Ge buffer layer, interface, Ge-selective epitaxial growth and layer quality. The photoluminescence (PL) of the samples was recorded using a 785 nm CW pumping laser, a liquid nitrogen cooled InGaAs detector. Hall measurements were performed at room temperature at a 0.5 T magnetic field using the standard Van der Pauw geometry pattern.

## 3. Results and Discussion

### 3.1. Growth Mechanism

**Figure 2:**
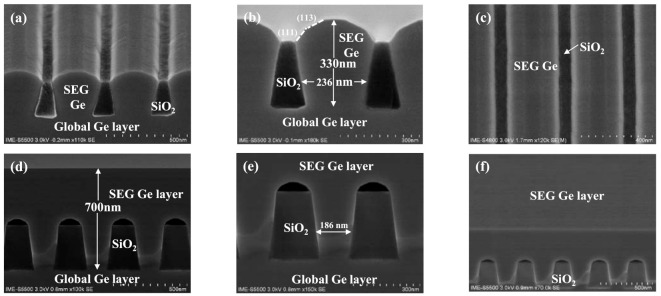
(**a**–**c**) SEM cross-section images of selectively grown Ge layer on patterned Ge-on-Si substrate with the aspect ratio of 1.36: (**a**,**b**) 400 nm SEG Ge; (**c**) top view of sample in (**a**) or (**b**); and (**d**,**e**) 700 nm Ge layer grown on pattern with the aspect ratio of 1.67; (**f**) tilted planar view of sample (**d**).

Recently, the “aspect ratio trapping (ART)” method has been intensively investigated to eliminate the threading dislocations in SEG of Ge [32,33]. Ge material was selectively grown in the groove where (111) and (113) facet planes were formed. In this epitaxy, the generated dislocations are depleted to oxide walls of the groove, causing the top layer to be grown with minor defects. In this experiment, a selective Ge layer was selectively grown in a trench in arrays with 220 nm and 180 nm width, which have aspect ratios of 1.36 and 1.67, respectively as shown in Figure 2. Initially, a 400 nm thick Ge layer was selectively deposited in 220 nm width trenches before the coalescence of growth fronts emerging from adjacent trenches as shown in Figure 2a–c. The cross-section SEM images show clear boundaries in different parts of the sample, showing no defects at the interface of global Ge buffer and selectively grown Ge layer. During SEG of Ge the whole trench has been uniformly filled, and the growth created (111) and (113) facets.

Later, a thicker Ge layer was formed where the lateral overgrowth with a thickness of 700 nm in 180 nm width trenches occurs as shown in Figure 2d–f. As the film thickness increases, Ge continues to grow along (111), (113) crystal plane direction until a continuous Ge layer with flat-top and symmetrical voids formed on the SiO_2_ mask as the result of the coalescence of Ge overgrowth in Figure 2e. Previously, some reports have demonstrated the formation of voids on SiO_2_ masks when a perfect coalescence is obtained [34,35]. The Ge overgrowth on the SiO_2_ surface occurs, and when the layer thickness exceeds a half of the SiO_2_ mask width, void formation occurs beneath the coalesced layer. Figure 2f shows the tilted view of selectively grown Ge layer with a mirror-like surface. This is mainly because of the semi-cylindrical void surfaces at the bottom can help the Ge layer to deplete TDs when the Ge overgrown layers coalesce but only when the TDD is large. However, if the TDD becomes smaller, voids cannot be used to remove isolated TDs. The mechanism of TDs formation and depletion has been previously reported [36]. It is important to point out here that aspect ratio trapping cannot, in itself, prevent TDs. Before coalescence of the Ge-pillars, the dislocations can be impeded to the side surfaces of the epi-pillar via aspect ratio trapping. However, isolated dislocations will reappear in the overlying material when adjacent epi-pillars coalesce into a continuous film. Therefore, “virtual dislocations” can be used as a visualization tool to enforce the rules governing dislocation topology during coalescence [36].

**Figure 3:**
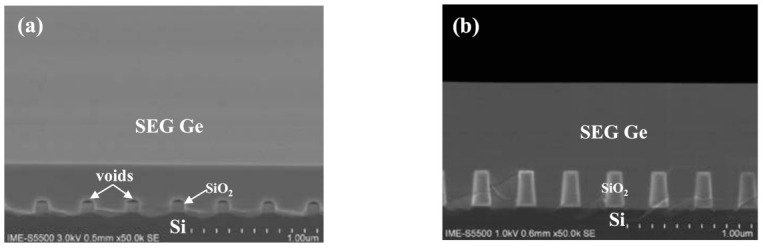
SEM cross–section images of selectively grown Ge layer with an aspect ratio of 1.67: (**a**) on a patterned Si substrate, and (**b**) sample (**a**) with post-annealing.

The formation of voids has been also observed in direct selective growth of Ge on Si substrates as shown in Figure 3a. The mechanism originates from the facet formation during the lateral overgrowth and continuing to coalesce Ge layer from sides. As will be discussed below in the XRD results, the presence of these voids has an important role in inducing compressive strain in Ge. Our analysis shows that an annealing treatment causes these voids to disappear, as shown in Figure 3b, and the strain is released.

**Figure 4:**
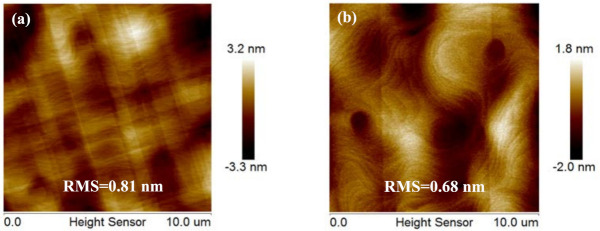
Two 10 × 10 μm^2^ AFM images of the Ge epilayers with: (**a**) globally grown layer; (**b**) se–lectively grown Ge layer on patterned Ge–on–Si substrate. The images show the 0.81 nm surface roughness for globally grown Ge and 0.68 nm for selectively grown Ge.

Figure 4a,b show the AFM images from the first 1400 nm global Ge growth on Si substrate and selective epitaxial growth of 700 nm Ge grown on 1200 nm patterned Ge-on-Si substrate. The root mean square (RMS), which is the indicator for roughness, was obtained at the value of 0.81 nm for 1400 nm globally grown Ge and 0.68 nm for selectively grown Ge surface on patterned Ge-on-Si substrate. The roughness of Ge layers is mainly generated due to the threading dislocation due to lattice mismatch, surface diffusion and thermal mismatch. We have demonstrated in our previous report that the surface roughness and TDD of a grown Ge layer on Si depend on its thickness [24]. When the thickness of Ge is increased, e.g., from 700 nm to 1500 nm, the surface roughness decreases (RMS of 0.81 nm), while for thicker layers, e.g., 2000 nm, the trend is reversed and the Ge quality layer is degraded (RMS of 1.03 nm). This problem is caused by the bowing of the wafer for thicker Ge layers. In this study, the above outcome was not observed and 700 nm selectively grown Ge on 1200 nm Ge-on-Si contains low defect density and low surface roughness, which is due to the defect depletion in the trenches.

**Figure 5:**
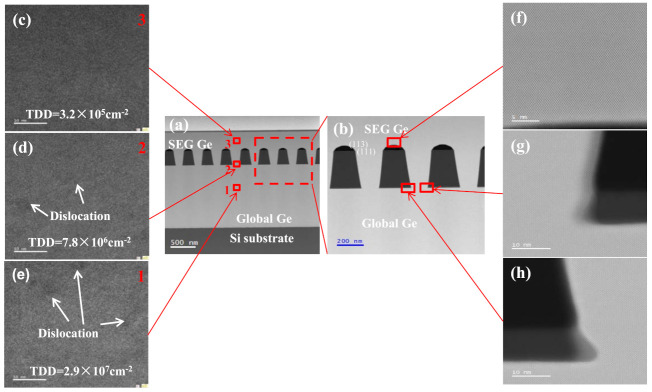
(**a**,**b**) Cross-sectional TEM image in the bright field of an SEG Ge/Ge/Si layer structure and (**c**–**e**) from different positions of (**a**): globally grown Ge layer, interface between selectively and globally grown Ge layers, and top Ge layer. They are the areas marked 1–3. (**f**,**g**) images show selectively grown Ge layer at the top, bottom, and corner of SiO_2_ mask.

In order to further analyze the crystal quality of epitaxial Ge in different positions in the samples, TEM analyses were carried out. Figure 5a shows a cross-section of TEM bright-field image of the Ge epilayer after overgrowth on the Ge-on-Si substrate. We selected the following three positions in the sample: globally grown Ge layer (area 1), interface between this layer and selectively grown Ge layer (area 2), and the top Ge layer (area 3) as shown in Figure 5a. The TEM images of positions 1 to 3 are zoomed in in Figure 5c–e. The 1400 nm global Ge layer contains few dislocations, and estimated TDD was about 2.9 × 10^7^ cm^−2^, according to TEM analysis. A minor number of dislocations were found at the interface between SEG Ge layer and global Ge layer, where the estimated TDD was about 7.8 × 10^6^ cm^−2^. In addition, no dislocation was observed in the selectively Ge layer in Figure 5c, and the estimated TDD value was as low as 3.2 × 10^5^ cm^−2^. It is worth mentioning here that our etch-pits experiments showed a higher TDD value of 2 × 10^6^ cm^−2^ for this sample. The deviation in TDD estimation could be due to the scale of measurements where etch-pits analysis has been performed in a larger crystal volume.

This AFM and TEM results indicate that the ART method can limit the threading dislocations and trap them by SiO_2_ sidewall leading to a high epitaxial Ge top layer on patterned Ge-on-Si substrate when the dislocation is large. Figure 5f,g shows TEM images of selectively grown Ge layer from several positions from top to bottom. It is clear that the atomic planes are well-arranged, and no dislocations were detected.

### 3.2. Strain Characterization

**Figure 6:**
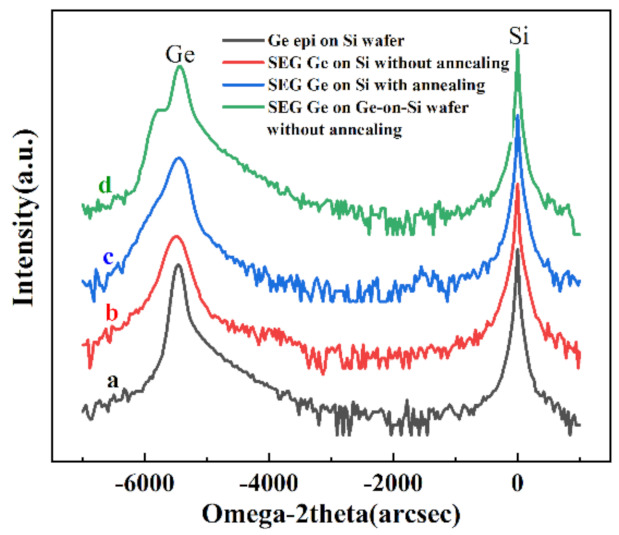
HRXRD (004) RCs of Ge layers: (**a**) global epitaxy of Ge on Si substrate; and (**b**) selectively grown Ge on patterned Si substrate, (**c**) sample in (**b**) after annealing, and (**d**) selectively grown Ge on patterned Ge–on–Si substrates.

Figure 6 illustrates HRXRD results from samples with Ge layers deposited either directly on Si or on Ge-on-Si substrates. In this series of samples, the curves are marked a to d in the Figure, and Ge-on Si (or bulk Ge) is considered as a reference sample. In general, the residual strain in thin films can come from three sources: (i) lattice misfit strain, (ii) thermal misfit strain, and (iii) defect strain. In these samples, tensile strain is initially induced in the Ge layer during cooling from growth or post-annealing temperature to room temperature due to different linear coefficients of thermal expansion (CTEs). The lattice distortion occurs in a perpendicular direction, and the lattice constant (*a*^⊥^) can be calculated by using Bragg’s law as follows:

(1) $$a^{⊥}=\frac{2\lambda }{sin\left(\frac{\omega }{2}\right)}$$

where *λ* is the wavelength of the incident radiation (Cu’s Κα1 line, *λ* = 1.5406 Å), and *ω* is the angular position of the Ge peak from the standard (004) *ω*-2*θ* scan. Using Equation (1), the *a*^⊥^ of the globally and selectively grown Ge layer can be estimated as 5.6472 Å and 5.6608 Å, respectively. The in-plane lattice constant (*a^||^*) of the Ge epilayer can be calculated using Equation (2) by considering the elastic modulus of Ge, *ν* = 0.271, and unstrained Ge lattice constant, α*_Ge_*= 5.6578 Å, as follows:

(2) $$\alpha^{\|}=\left(\frac{1+v}{v}\right)\left[\alpha_{\mathrm{Ge}}-\alpha^{⊥}\left(\frac{1-v}{1+v}\right)\right]$$

Therefore, the estimated *a*^||^ of global Ge layer and SEG Ge layer samples are 5.6722 Å and 5.6518 Å, respectively. The residual strain of the Ge epilayers can be calculated from Equation (3):

(3) $$\varepsilon =\frac{\alpha^{\|}-\alpha_{\mathrm{Ge}}}{\alpha_{\mathrm{Ge}}}\;\%$$

Positive and negative values of *ε* for the Ge epilayer indicate either tensile and compressive strain.

Figure 6d shows two Ge peaks appearing in the curves, one on the left side and the other one on the right side of the main Ge peak showing compressive and tensile strain, respectively. The tensile Ge peak is not well distinguished, and it merely appears in an elongated feature in Figure 6a,d. The maximum amount of this tensile is about 0.25%, while the maximum formed compressive strain is −0.12% in the selectively grown Ge-on-Si in Figure 6d. It is believed that the compressive strain is induced in the presence of voids in lateral overgrowth of Ge, as it is shown in Figure 2d,e. However, there are voids in SEG Ge on Si in Figure 6b, but the amount of the compressive strain is remarkably lower than the selectively grown Ge layer on Ge in Figure 6d. The other observable point is the different Full-Half-of-Maximum (FWHM) of Ge peak in curves from Figure 6a–d. The FWHM of the x-ray peak increases for thinner layer and/or higher amount of defect density. Firstly, it is difficult to estimate the FWHM in Figure 6d since there are two closely situated Ge peaks. Meanwhile, in these samples, the thickness of selectively grown Ge-on-Si (in Figure 6b) (in Figure 6c) was about 1 µm compared to 0.7 µm for selectively grown Ge-on-Ge (in Figure 6d). Therefore, broader FWHM is expected in the Ge-on-Ge sample compared to the Ge-on-Si sample, but the Ge-on-Si sample has a broader FWHM due to the higher defect density. The FWHM for this sample in Figure 6c is slightly improved after annealing treatment.

From the calculations, the values of *ε* are 0.25% for globally grown Ge epilayer and –0.12% for selectively grown Ge layer. In other words, we can obtain tensile strain for globally grown Ge epilayers but compressive strain for selectively grown Ge layer. This is an impressive result that compressive and tensile strain is introduced in Ge in different positions in one wafer at the same time.

**Figure 7:**
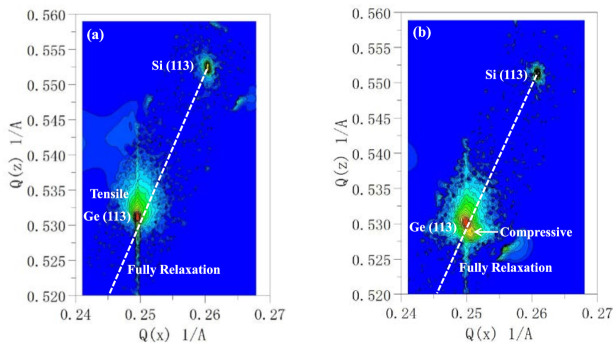
HRRLMs around (1 1 3) reflection for the (**a**) global Ge layer on Si substrate and (**b**) SEG Ge layer on patterned Ge–on–Si substrate.

In order to confirm the strain profile in these samples, high-resolution reciprocal lattice maps (HRRLMs) around (113) reflection were performed, as shown in Figure 7a,b. It is confirmed from Figure 7a that the globally grown Ge layer was tensile-strained, whereas the second sample with globally and selectively grown layers contained tensile strain and compressive strain layers from the two peaks in Figure 7b. The results are consistent with the above HRXRD, revealing that tensile and compressive strain exist at different positions in one sample.

**Figure 8:**
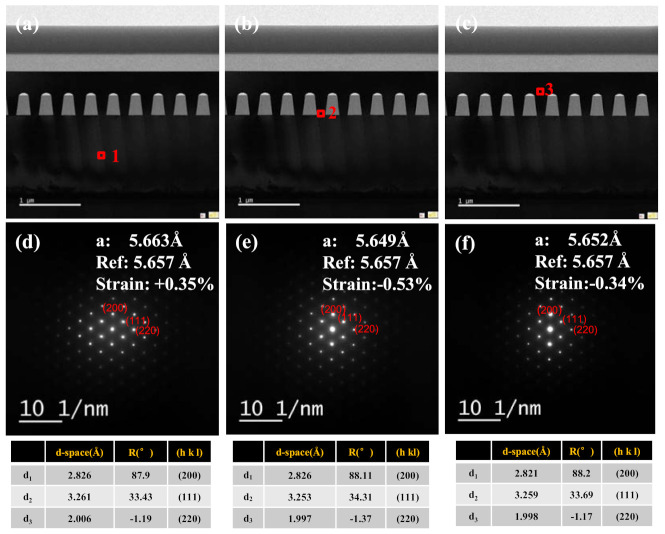
Cross-section HRTEM images of the Ge epilayer of (**a**) globally grown Ge layer, (**b**) interface of Ge layer, and (**c**) Ge top layer. The electron diffraction patterns obtained for different regions are (**d**–**f**).

In order to further analyze the strain characteristics in different positions in Ge layers, the electron diffraction pattern of TEM was needed. Figure 8 shows the selected-area electron diffraction (SAED) patterns from different 1, 2 and 3 marked areas in the Figure displaying good single-crystalline features of these layers. In order to calculate lattice spacing d in different positions, the following formula is applied:

*Rd* = *Lλ* (4)

where *R* is the length of the camera, *λ* is the wavelength of the incident radiation and *R* is the spacing of the crystal planes corresponding to the diffraction bands. The *d-values* of the above three points are listed in the tables below each image. Then, the lattice constant *a* can be derived from the different lattice spacings *d*_1_, *d*_2_ and *d*_3_.

The lattice constant values in 1, 2 and 3 positions in the Ge layers are 5.663 Å, 5.649 Å and 5.652 Å. Compared with the standard Ge lattice constant 5.6578 Å, the strain of the globally grown Ge layer, Ge interface and selectively grown Ge layer are 0.35%, −0.53% and −0.34%, respectively. This result shows that there is an initial tensile strain in the globally grown Ge layer, but it becomes a compressive strain along the SiO_2_ trench in the [001] direction. The change in the strain along the trench to the upper layer is due to the relaxation of Ge along the trench and in presence of the voids.

According to the growth mechanism of heterostructures, the strain due to lattice mismatch is expected to be fully relaxed by misfit dislocations that nucleate at the free surface. These dislocations glide and propagate to the interface when the critical thickness of Ge on Si is~1 nm in the range of our growth temperature [37]. Then, the induced strain due to the thermal mismatch for growth carried out at 923 *K* followed by cooling to 298 *K* is given:

(5) $$\varepsilon^{\|}=\int_{25}^{650}[\alpha_{\mathrm{Ge}}\left(T\right)-\alpha_{\mathrm{Si}}\left(T\right)]\mathrm{dT}$$

Where α_Si_ (2.6 × 10 ^−6^ *K*^−1^) and α_Ge_ (5.8 × 10^−6^ *K*^−1^) are the thermal expansion coefficients of Si and Ge, respectively, and *ε*^‖^ is the strain parallel to the interface. This strain relaxation involves nucleation and glide of dislocations in a relatively thick film, so the strain components *ε*^‖^ and *ε*^⊥^ are related by [38,39]:

(6) $$\varepsilon^{⊥}=\frac{-2\upsilon }{\left(1-\upsilon \right)}\;\varepsilon^{\|}$$

Using Equation (5), the strain of the global Ge layer was calculated to be +0.21% (tensile strain). However, for the SEG Ge layer, since it is a homogeneous epitaxial on groove patterned Ge/Si substrate, there is no thermal mismatch strain interface in the parallel direction. Meanwhile, for Ge layer grows in trench SiO_2_, there will be thermal-induced strain along the trench direction due to the mismatch of thermal expansion coefficient between Ge and SiO_2_ (0.5 × 10^−6^ *K* ^−1^); therefore, we should calculate perpendicular strain *ε* for the selective grown Ge layer. Similarly, using Equations (5) and (6), the perpendicular strain *ε*^⊥^ of this layer is estimated to −0.25%. However, according to TEM analysis, the value of the compressive strain decreases gradually along the direction of the groove, and the maximum strain (−0.53%) exists at the bottom of the trench. This is due to the confinement of Ge layer surrounded by the SiO_2_ and Ge at the side wall and the bottom. In addition, in our experiments, ART technology can limit the movement of the threading dislocation on the glide planes of (111), (113) by the SiO_2_ wall. Therefore, the value of compressive strain decreased along the trench’s parallel direction.

**Figure 9:**
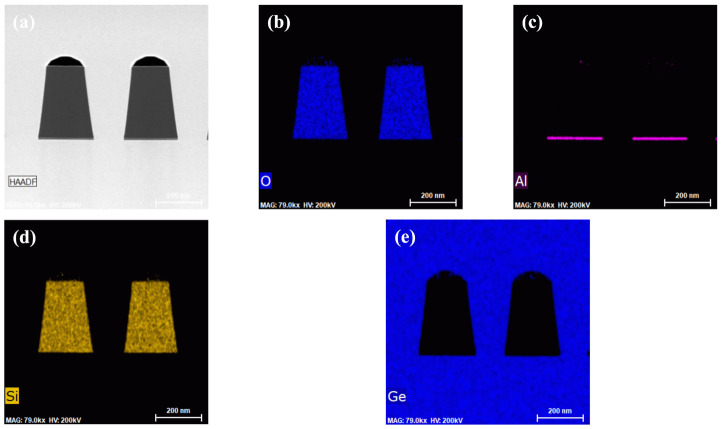
(**a**) Cross–sectional TEM image of the SEG Ge/Ge/Si layer structure, EDS mappings of the SEG Ge regions with elements of (**b**) O, (**c**) Al, (**d**) Si and (**e**) Ge.

Figure 9 shows the element analysis at areas of the sample 700 nm SEG Ge layer on patterned Ge-on-Si substrate. The results show that the boundary of each layer was consistent with the designed structure, and there was no obvious diffusion of elements. The results provide an experimental basis for manufacturing high-purity Ge materials for the optoelectronic device in the future.

**Figure 10:**
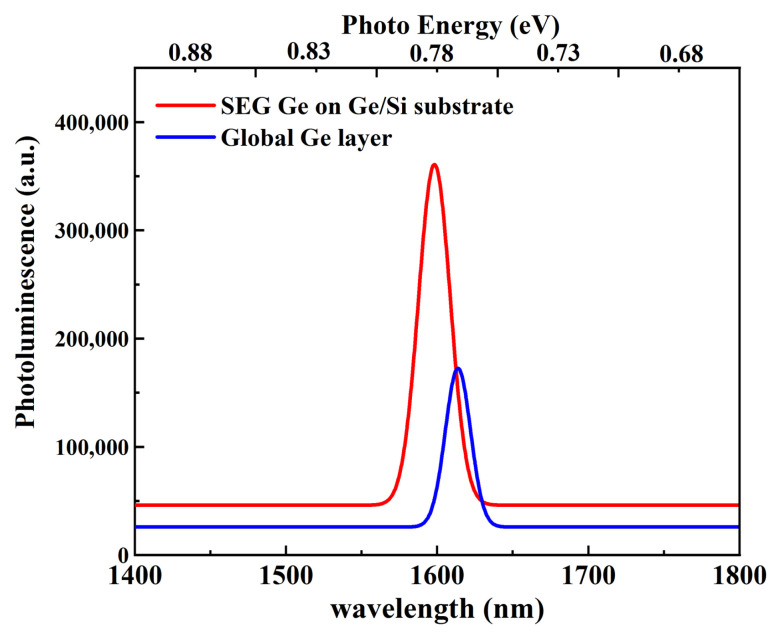
Room–temperature PL spectra for the global Ge layer and SEG Ge on Ge/Si substrate.

In this work, the final analysis of selectively grown Ge layers was performed by the PL technique, which is very sensitive to the presence of defects. In general, there are several factors that influence the PL intensity, e.g., threading dislocation, surface scattering, recombination center caused by diffusion and surface non-radiative recombination centers. The surface roughness and various crystal defects cause the internal recombination sites, leading to a sharp decrease in the PL intensity. Figure 10 shows the PL characterization of the Ge layers carried out at 298 *K* using a 785 nm CW pumping laser. In these spectra, PL of the selectively grown Ge layer has a stronger intensity (three times greater) than that of the globally grown Ge layer. This may be attributed to the following two reasons: (i) the decreasing in recombination centers as a result of the low TDD and (ii) the efficiency improvement of light collection due to the SiO_2_ trench and the top voids as the reflective layers. Low defect density and interface diffusion can reduce carrier recombination, which leads to the high PL intensity for selectively grown Ge layer.

In our experiments, Hall mobility measurements were also made for the above two samples at room temperature. The results show that the hole mobility of the selectively grown Ge layer was 375 cm^2^/Vs compared to the globally grown Ge layer with 132 cm^2^/Vs. The results of PL and Hall measurement are consistent with the previous TEM analysis results: the selectively grown Ge layers have excellent crystal quality.

Table 1 shows the strain data extracted by different measurements. It can be confirmed that tensile strain and compressive strain exist at the same time in one wafer. However, the compressive strain values calculated from HRXRD and TEM are somewhat different. This is attributed to the different measurements: HRXRD tests the value in a global way, but TEM tests the value in local areas. Ge PL position can further verify the trend of the strain. At the same time, TDD calculation results also show that SEG Ge has a better crystal quality.

## 4. Conclusions

We have demonstrated selectively grown Ge layers with compressive strain showing high layer quality and excellent electrical transport. The fabrication procedure is composed of global tensile-strained Ge epilayer growth followed by CMP treatment to planarize the Ge surface. Later, SiO_2_ layers were deposited and were patterned for SEG of Ge layer. The structures were analyzed by TEM, HR-XRD, PL and Hall measurements to discover the defect density, strain and carrier mobility in the Ge layers. In contrast to the global tensile strained Ge layer, enhanced PL intensity by a factor of more than 2 is partially due to the improved material quality. SiO_2_ trenches are regarded as the reflector for PL emission, which also has the contribution to the higher PL intensity. Hall measurement shows that the compressive strained Ge layers possess hole mobility up to 375 cm^2^/Vs at room temperature, which is approximately 3 times larger than that of the global Ge layer (132 cm^2^/Vs). This work provides the fundamental guidance to fabricate high-quality Ge epilayer on Si with high compressive strain.

## Figures and Tables

**Table 1:** PL positions and strain data calculated from HRXRD and TEM plus threading dislocation densities for each sample.

Sample	Strain Calculated by HRXRD	Strain Calculated by TEM	Ge PL Position	Extracted TDD in Ge by TEM (cm^−2^)
Global Ge	+0.25%	+0.35%	0.768 eV	2.9 × 10^7^
SEG Ge	+0.25% −0.12%	+0.35% −0.34%	0.781 eV	3.2 × 10^5^

## Data Availability

The data presented in this study are available on request from the corresponding authors.
